# Mit den Augen Susan Sontags: Metaphern im Umgang mit COVID-19

**DOI:** 10.1007/s42048-021-00098-4

**Published:** 2021-04-26

**Authors:** Henriette Krug

**Affiliations:** University of Applied Sciences and Medical University, Fakultät Gesundheitswesen, Medical School Hamburg, Am Kaiserkai 1, 20457 Hamburg, Deutschland

**Keywords:** Pandemie, Covid-19, Metapher, Lebenshaltung, Krankheit, Gesundheit, Pandemic, Covid-19, Metaphor, Attitude towards life, Illness, Health

## Abstract

In der Erfahrung, Kommunikation und Bewältigung von Krankheit spielen Metaphern eine wichtige Rolle: Als Denkkonzepte spiegeln sie zugrundeliegende Haltungen gegenüber den durch sie beschriebenen Vorgängen wider. Susan Sontag hat mit ihrem Essay „Illness as Metaphor“ nachhaltig die moralisch kritischen Implikationen einer unreflektierten Metaphernverwendung im Umgang mit Erkrankung aufgezeigt, indem sie deren stigmatisierende und hierin zusätzlich belastende Wirkung für Erkrankte reflektiert.

In der gegenwärtigen Situation der Covid-19-Pandemie trifft ein bisher unbekanntes Virus mit der Macht und Dynamik der Globalisierung auf die hierauf nicht vorbereiteten spätmodernen Gegenwartsgesellschaften, die nun nach Erklärung und Sinnzuschreibung suchen. Auch hier fungieren Metaphern als Denkkonzepte und Vehikel von Interpretation. Im Rückgriff auf die Perspektive Susan Sontags und ihren Appell für einen entmystifizierten Umgang mit Krankheit geht dieser Beitrag folgenden Fragen nach: Welche Bilder werden in der Berichterstattung über CoV‑2 vermittelt? Welche Einstellung gegenüber Gesundheit und Krankheit wird mit den Metaphern transportiert, und welche Lebenshaltung liegt dieser zugrunde? Welche Einsichten für einen gesunden Umgang mit Covid-19 und Krankheit generell ergeben sich, wenn man die Pandemie aus ihren Metaphern und zugrundliegenden Denkkonzepten herausschält?

## Abstract (en)

Metaphors play an important role in the experience, communication and management of diseases: They are a constitutive psychological tool in understanding complex issues and they influence the cognitive conceptual structures we use in order to interpret them. In her powerful essay “Illness as Metaphor” Susan Sontag reflects on the moral implications of an uncritical use of metaphors for diseases that may disgrace and stigmatise, and thus evoke additional burden, to sick persons.

In the current situation of the Covid-19 pandemic, metaphors are used as means of explanation and interpretation, too. By adopting Sontag’s demystifying perspective, the essay raises the following questions: Which metaphors are used in the public media for describing Covid-19? Which underlying attitude towards health, illness and life is communicated through this use? Regarding a healthy, rational handling of Covid-19 as well as illness in general: What insights will remain if we dispel metaphors and their underlying modes of thinking?

## Einführung: COVID-19 mit der Perspektive Susan Sontags

### Fragestellung: Metaphernbildung bei COVID-19

Aktuell steht die Welt unter dem Eindruck der COVID-19-Erkrankung (Covid-19), ausgelöst durch das Virus SARS-CoV‑2 (CoV-2). Die Menschen der spätmodernen Gegenwartsgesellschaften erleben sich mit einer Gefahr für ihr Leben konfrontiert, auf die sie mental wie institutionell nicht vorbereitet waren: Ein mikroskopisch kleines Virus breitet sich strukturanalog zu der Effizienz und Geschwindigkeit der global vernetzten Welt grenzüberschreitend aus, hebelt grundsätzliche Gewissheiten und Routinen aus und legt weltweit den Alltag lahm. Dieses Geschehen wird intensiv wissenschaftlich wie gesellschaftlich kommuniziert und diskutiert. Hierbei sind in vielen Kommunikationsbereichen Metaphern als Denkkonzepte und Vehikel von Interpretation im Einsatz. Susan Sontag schrieb 1978 mit ihrem Essay „Krankheit als Metapher“^1^ einen bis heute vielzitierten Text, in dem sie sich kritisch mit den Auswirkungen einer metaphorischen Deutung und Überhöhung von Krankheit auseinandergesetzt hat. Angestoßen durch diese Perspektive soll in diesem Beitrag die Metaphorik in der gegenwärtigen medialen Diskussion im Zusammenhang mit Covid-19 vorgestellt und daraufhin befragt werden, welche Anliegen und Affekte und welche grundlegende Einstellung der Gesellschaft gegenüber Gesundheit und Krankheit darin transportiert werden.^2^ Im Hinblick auf die Wirkmacht von Metaphern schließt sich eine kritische Reflexion an: Insofern Gesundheits- und Krankheitserleben eng verknüpft sind mit dem Lebensstil und der Frage nach dem guten oder gelingenden Leben, d.h. der Frage, „wie zu leben ist“ (Steinfath 1998:14), ermöglicht die genauere Betrachtung der Metaphorik, die eine Gesellschaft in diesem Zusammenhang verwendet, das Erkennen wie das kritische Reflektieren der Denkweisen über die hierin kommunizierten Bedingungen guten bzw. gesunden Lebens. Dies führt entlang der Perspektive Susan Sontags zur abschließenden Frage, welche Einsichten für einen gesunden Krankheitsumgang sich ergeben, wenn man die Pandemie aus ihren Metaphern und zugrunde liegenden Denkkonzepten herausschält.

### Krankheit und Metapher

Metaphern begegnen ubiquitär im menschlichen Sprachgebrauch. Gemäß der an die aristotelische Metapherndefinition anknüpfenden Substitutionstheorie besteht eine Metapher im Austausch eines Wortes durch ein fremdes Wort und stellt eine Verwandtschaft her zwischen dem mit dem Wort ursprünglich bezeichneten und dem metaphorisch beschriebenen Objekt (Kurz 2009). An seinem neuen Ort ersetzt und illustriert dieses das ursprüngliche Wort, oder es bildet einen Begriff für Dinge, für die eine eigentliche Formulierung lexikalisch fehlt. Nach interaktionstheoretischen Ansätzen ist bei der Verwendung von Metaphern über dieses Verständnis einer rein sprachlichen Ausdrucksform hinausgehend zudem deren Kontextualität und Situativität zu berücksichtigen. So sind sie immer nur als Teil und Funktion der jeweiligen Situation zu verstehen, in die hinein sie gesprochen werden: „Es gibt keine sprachliche Bedeutung an sich, sondern nur in bestimmten Situationen, für bestimmte Sprecher und Hörer, für bestimmte Absichten. Wenn wir uns die Bedeutung eines Wortes klar machen, müssen wir uns seine Verwendung klarmachen.“ (Kurz 2009: 14) Anliegen und Wirkung des Metapherneinsatzes bestehen somit nicht nur darin, illustrativ bestimmte Bilder oder Assoziationen vor dem inneren Auge hervorzurufen und so Kommunikation reicher und effizienter zu gestalten, sondern vielmals „vor allem darin, eine effektive Einstellung zu erzeugen“ (Kurz 2009: 26). Lakoff und Johnson beschrieben Metaphern als Denkkonzept und psychologisches Phänomen, das menschliche Kognitionsprozesse und -strukturen sowohl spiegelt als auch formt (vgl. die „conceptual metaphor theory“, CMT, in Lakoff und Johnson 1980). Demnach sind Metaphern konstitutiv für das Denken und Verstehen abstrakter und komplexer Themen und haben hohes Erklärungs- und Überzeugungspotenzial in vielschichtigen Zusammenhängen. So sind sie z.B. im Kontext von wissenschaftlicher Theoriebildung und Fachsprache ebenso ein bewährtes Denk- und Kommunikationsmedium wie in der Wissensvermittlung an Laien (vgl. Biere und Liebert 2013). In einer Linie mit dieser Theorie konnte in Studien gezeigt werden, dass unsere Denkprozesse durch den Einsatz von Metaphern beispielsweise im Umgang mit sozialpolitischen Fragestellungen, Krankheitserleben oder emotionalen Erfahrungen beeinflusst werden, ebenso unsere Grundeinstellung und Entscheidungsprozesse (Thibodeau et al. 2019). Somit ist davon auszugehen, dass Metaphern eine wichtige Rolle zukommt für die Art und Weise, wie wir kulturspezifisch neue, komplexe Themen aufnehmen und verarbeiten (vgl. z.B. Musolff 2012). Andersherum kann die Untersuchung der angewandten Metaphorik Einsicht vermitteln in die zugrundeliegenden kognitiven Prozesse in der Wahrnehmung der Lebenswirklichkeit.

Krankheit ist ein mehrdimensionales Geschehen und hat immer auch eine soziale Gestalt. In ihrer sozialen Gestalt werden Krankheitsbilder über ihre bloße physische Erscheinungsform hinaus in ihrer Auswirkung und Bedeutung für die betroffenen Individuen wie ihr Sozialgefüge gedeutet (vgl. z.B. Hanses und Richter 2011). Dabei bietet das Erleben von Krankheit ein weites Feld für Projektionen, in deren Beschreibungen Metaphern ein wichtiges Vehikel für Deutungsmuster bilden.

Die wissenschaftliche wie gesellschaftliche Kommunikation über Krankheit, Gesundheit und Medizin, ebenso auch konkrete Gesprächssituationen zwischen Arzt und Patient, sind dementsprechend reich an Metaphern (vgl. z.B. Hodgkin 1985, Schachtner 2001, Kamps 2004, Bauer 2006; Marron et al. 2020). Sie transportieren sowohl die je kulturell geprägten Vorstellungen und Erlebnisqualitäten von der Erkrankung selbst als auch Bilder von den durch sie ausgelösten Emotionen, Phantasien und Anliegen. So vermitteln sie implizit Einsicht in die Denkweise, mit der Menschen das Themenfeld Krankheit und Gesundheit angehen und damit einen Einblick in die zugrundeliegende Lebenseinstellung. Insbesondere im Bereich der narrativen Medizin besteht ein hohes Bewusstsein für die grundlegende und konstruktive Bedeutung einer metaphorischen Verständigung (Coulehan 2003, Kamps 2004). So kann eine reflektierte Metaphernverwendung positiv zum Verstehen wie Verarbeiten von Krankheit beitragen und sich so gesundheitsförderlich auswirken (Schachtner 2001). Ebenso wissen wir, dass – insbesondere bei unbewusst eingesetzten Metaphern – negative Implikationen hervorgerufen werden können, so dass ein sprachsensibler Einsatz geboten ist (Hodgkin 1985, Coulehan 2003). Dies gilt auch im Hinblick auf ihre kulturelle Kontextualität und die hiermit gegebene Anfälligkeit für Missverständnisse (Masukume und Zumla 2012). Marron et al. (2020) analysieren das zwiespältige Potenzial der im onkologischen Kontext vielfach verwendeten Kriegs- bzw. Kampfesmetaphorik: Neben positiven Aspekten wie Verständlichkeit, Ausdruck der Dringlichkeit, Handlungsfähigkeit und möglicher Sinnstiftung sind bei der Rede vom Krieg oder Kampf gegen eine Erkrankung auch negative Effekte wie die implizite Eröffnung der Kategorien von Sieg und Niederlage (die Genesenden als Sieger, die Kranken als Verlierer) oder unangebrachte Sorglosigkeit zu berücksichtigen, wenn unter dem Eindruck dieser Metapher vom Vorhandensein bereits wirksamer Kampfesstrategien und -ausstattung ausgegangen wird. Im Hinblick auf die onkologischen Erfahrungen mit der Verwendung dieser Metapher ist das Urteil ambivalent: „Use war metaphors with caution; they are an ethical minefield“ (Marron et al. 2020: 2).

Susan Sontag schrieb mit ihrem Essay „Krankheit als Metapher“ einen nachhaltig wirksamen metaphernkritischen Text über die sozial-gesellschaftliche Dimension von Krankheit. Darin zeigte sie die jeweiligen Gefühlsphantasien und sprachlichen Bilder auf, die sich historisch in der Literatur mit dem Begriff von Krankheit verbinden und in den sozialen Umgangsformen mit bestimmten Erkrankungen – dargelegt anhand der Diagnosen Tuberkulose und Krebs – mitteilen. Sie konstatierte eine metaphysische und moralische Aufladung von Erkrankungen, insbesondere von solchen, die als medizinisch noch unklar und nicht heilbar wahrgenommen werden, und erklärte diese menschliche Neigung vor dem Hintergrund der Grundprämisse einer allheilenden Medizin. Solange eine Krankheit als unverstanden und als nicht therapierbar gelte, werde sie als unheimlich und geheimnisvoll mystifiziert: „Eine solche Krankheit ist per se mysteriös.“ (Sontag 2016:10) In ihrem Text fokussiert sie auf die Gefahr solch einer metaphysischen Überhöhung und den hiermit oftmals verbundenen Straf- und Schuldzuweisungen an von Krankheit Betroffene. Sie legt die suggestive wie schädigende Wirkung eines unreflektierten Metapherngebrauchs in Form von zusätzlicher Stigmatisierung und moralischer Belastung dar und tritt ihr entgegen, indem sie die den Metaphern zugrundeliegende und diese formende Denkweise gegenüber Medizin und Krankheit aufdeckt und kritisiert: „Solange eine besondere Krankheit als ein bösartiger, unbezwingbarer Feind und nicht einfach als Krankheit behandelt wird, werden die meisten Menschen mit Krebs in der Tat demoralisiert sein, wenn sie erfahren, was für eine Krankheit sie haben“ (2016:11). Mit ihrem Essay versucht sie, diese Verbindung von Krankheit und Metapher zugunsten eines unverstellten Blickes und gesunderen Umganges mit einer Erkrankung aufzubrechen: „Zeigen will ich, daß Krankheit *keine* Metapher ist und die ehrlichste Weise, sich mit ihr auseinanderzusetzen – und die gesündeste Weise, krank zu sein –, darin besteht, sich so weit wie möglich von metaphorischem Denken zu lösen, ihm größtmöglichen Widerstand entgegenzusetzen.“ (Sontag 2016: 9).

Blieb Sontags kritische Auffassung der Metaphernverwendung zwar ihrerseits keineswegs unwidersprochen (vgl. z.B. Clow 2001 oder Coulehan 2003), so hat sie in jedem Fall nachhaltig das Bewusstsein für einen sprach- und kultursensiblen Umgang mit sprachlichen Bildern im Zusammenhang von Krankheit, Gesundheit und deren Deutung geschärft. Zusammengefasst wird angesichts der prägenden wie persuasiven Macht von Metaphern und der von Susan Sontag aufgezeigten potenziell schädigenden Auswirkungen von metaphorischer Rede über Krankheit deutlich, wie wichtig es ist, Sprachbilder im Bereich von Gesundheit und Medizin kritisch zu reflektieren und solchermaßen sensibilisiert einzusetzen, dass schädigende Konsequenzen im Umgang mit Kranken vermieden werden, gesundheitsförderliche Implikationen sich aber voll entfalten können.

## Metaphern zu Covid-19: Welche Grundhaltung und Anliegen werden erkennbar?

In der medialen Kommunikation über CoV‑2 und seine Auswirkungen auf die Lebenswirklichkeit wird eine Vielzahl von Metaphern in unterschiedlicher, sich gegenseitig beeinflussender Funktion verwendet. Als ein zunächst primär naturwissenschaftliches Themengebiet sind in der Berichterstattung über CoV‑2 im weiteren Sinne wissenschaftsjournalistische Inhalte aus Biologie, Infektiologie und Epidemiologie zu transportieren. Im Wissenschaftsjournalismus fungieren Metaphern als etablierte „probate kognitiv-sprachliche Mittel der Wissensvermittlung“ (Bischl 2013: 103). Sie werden hier bewusst als Erklärungsmodelle und Denkkonzepte zur Unterstützung von Verstehensprozessen eingesetzt und nehmen dabei ggf. Sprachbilder auf, die im wissenschaftlichen bzw. medizinisch-ärztlichen Kontext bereits verwendet werden. Bei der Beschreibung der psychosozialen, (gesundheits)-politischen und sozioökonomischen Implikationen innerhalb der Gesellschaft kommt Metaphern neben dieser Erklärungsfunktion zusätzlich eine Spiegel- wie Prägefunktion zu: sie reflektieren einerseits die Wahrnehmung und Erlebnisqualität des Pandemiegeschehens innerhalb der Bevölkerung, indem sie dort kursierende interne Auffassungen und Interpretationen aufgreifen und kommunizieren, prägen aber andererseits auch zugleich Haltungen und Einstellungen in menschlichen Denkprozessen (Thibodeau et al. 2019), wenn sie als externe Interpretationsmodelle aufgenommen und internalisiert werden. Insofern werden die im Folgenden aufgeführten Metaphern als Ausdrucksform eines medial vermittelten wechselseitigen Prozesses aus externen Erklärungs- und Interpretationsmustern und internen Denk- und Verarbeitungskonzepten verstanden, die als situative individuelle wie kollektive Verarbeitungskonstrukte die Sicht- bzw. Verhaltensweisen der Gesellschaft im Umgang mit der Pandemie spiegeln, formen und auch antizipatorisch bestimmen.

### Die Welt wird angegriffen: Covid-19 als ungeheuerlicher Gegner

In der gesellschaftlichen, politischen aber auch fachlich-medizinischen Berichterstattung über CoV‑2 begegnet vielfach die Metaphorik von Angriff 3, Krieg^4^ und Kampf 5. Dementsprechend erscheint in verschiedenen medialen Darstellungen und Zitaten das Virus als eine mikroskopisch kleine Macht, die heimtückisch in einem Zero-Day-Angriff weltweit die unvorbereitete Menschheit befällt und zur globalen Gefahr wird. Die sich verbreitende Krankheit ist neu: Der gegnerische Eindringling CoV‑2 kann diverse bedrohliche Symptome hervorrufen, auf individuell-körperlicher Ebene vor allem Atemnot bis hin zum Tod, auf globaler Ebene Stillstand und wirtschaftlich-sozialen Notstand. Weitere unheimliche Kennzeichen sind die hohe, unkontrollierte Geschwindigkeit seiner Ausbreitung. Die Menschen erleben hierin einen sich unaufhaltsam verbreitenden Verlust der Kontrolle über ihre bisher wahrgenommene gesundheitliche Sicherheit und Routine. Als akute Reaktion resultieren Verunsicherung und Angst, verbunden mit dem Ruf nach Abwehr und Verteidigung^6^: „Mehr Soldaten im Kampf gegen Corona in Berlin.“^7^ Es gilt, mit Forschungsprojekten und gesundheitspolitischen Strategien das Virus in seiner aggressiven Infektiosität zu bekämpfen, um die Kontrolle über den Gesundheitsstand der Bevölkerung zurückzugewinnen.^8^ Doch solange geeignete Waffen wie Impfstoff oder passender Virustatika fehlen^999^, drängt sich das Bild eines ungeheuerlichen, weil übermächtigen Gegners auf.^9^

Gemäß Sontags Erklärung muss das Coronavirus geradezu als Einladung zu metaphorischer Ausdeutung und Überhöhung wirken, da es nicht nur als bisher unbekannt und untherapierbar gilt, sondern durch seine globale Verbreitung zudem ein „kollektives Unglück“ (Sontag 2016: 52) darstellt und zusätzliches Gewicht erhält. Den Beschreibungen von CoV‑2 als gegnerischem Angriff auf die Gesundheit und das Leben der Menschen korrespondiert der Kampfaufruf zur Verteidigung. Die Angegriffenen treten aus der passiven Opfer- in die aktiv-kämpferische Rolle mit dem Ziel, die Kontrolle über ihre gesundheitliche Sicherheit zurückzugewinnen.^10^ Neben dem Verteidigungswunsch gegenüber der vitalen Gefahr wird – wie bereits Sontag als Grundprämisse der Metaphernbildung über Krankheiten herausstellte – deutlich, wie auch heute die Überzeugung von einer überaus erfolgreichen Medizin Urheberin metaphorischer Deutung ist. Die Metaphern von Kampf und Krieg zeigen die angesichts der Unterminierung dieser Prämisse empfundene Aggression wie Dringlichkeit einer Entgegnung: Bis dato prägte der Eindruck einer anhaltenden Erfolgsgeschichte mit ständiger Expansion von Wissen und Therapieerfolgen die gesellschaftliche Perspektive auf Medizinwissenschaften, Gesundheit und Krankheit. Das kontinuierlich zunehmende Potenzial der Medizin, Krankheiten in ihrem Auftreten kontrollieren und verhindern zu können, einhergehend mit dem seit Jahren messbaren Ansteigen von Gesundheitsstandards und Lebenserwartung in der Bevölkerung, hat allgemein die Vorstellungskraft gemindert, im Leben mit dem Auftreten von schwerer Krankheit zu rechnen und die Bereitschaft gesenkt, diese im eigenen Lebensentwurf zu tolerieren. Das gilt besonders im Hinblick auf Infektionskrankheiten, deren Gefährdungspotenzial für die Industrienationen durch die dortige Verfügbarkeit zahlreicher suffizienter Pharmaka extrem gesunken ist.

Nicht nur die Perspektive auf Krankheit und Gesundheit ist geprägt von Kontroll- und Verfügbarkeitsdenken, auch der Blick auf das Ende menschlichen Lebens: Die Intensivmedizin vermag auf vielfache Weise in den Sterbeprozess einzugreifen und den Todeseintritt zu verzögern oder gar zu verhindern. Die meisten Menschen sterben in medizinischen Einrichtungen, d.h. der Sterbeprozess ist von Hospitalisierung und Medikalisierung gekennzeichnet und kontrollier- wie verhandelbar geworden.^11^ In der offiziellen Einteilung von Sterblichkeitsursachen in der Gesundheitsberichterstattung des Bundes bildet sich dieser Gedanke der Verhandelbarkeit des Todes in der Kategorie vermeidbarer Sterbefälle ab: „Als vermeidbar werden vielfach Sterbefälle mit ausgewählten Todesursachen bezeichnet, von denen angenommen wird, dass sie (in einem bestimmten Altersfenster) bei angemessener Behandlung und Vorsorge im Prinzip hätten verhindert werden können.“ (Gaber und Wildner 2011: 43). Die derzeit mit den Präventionsmaßnahmen einhergehenden Einschränkungen des öffentlichen wie privaten Lebens werden mit der Verminderung bzw. Vermeidung weiterer Infektionen durch Cov‑2 begründet, ein Sterben an Covid-19 gilt damit als verhinderbar. In diesen gesundheitspolitischen Kategorien zum Sterben wird zusätzlich deutlich, in welchem Maß Leben und Gesundheit als selbstverständlich verfügbar und modulierbar angesehen werden. „Gesundheit und Krankheit erscheinen der modernen Medizin immer weniger als Geschicke, als Gegebenheiten, sondern immer mehr als Resultate der eigenen Handlungen, ja als Erzeugnisse des eigenen Willens.“ (Maio 2014: 20)

Die im Selbstverwirklichungs- und Optimierungsdenken verortete Rede vom gelingenden Leben katalysiert diese Sichtweise zusätzlich, da Gesundheit hierfür als unverzichtbare Grundvoraussetzung gilt, Krankheit hingegen als Entwertung oder Diebstahl (Schneider-Flume 2002: 85 ff.). „Der moderne Mensch ist fest davon überzeugt, dass es nichts gibt, womit man sich heute abzufinden hat.“ (Maio 2014: 13) Die frühere Jahrhunderte prägenden Gedanken von einer grundsätzlichen Unverfügbarkeit oder einem Geschenkcharakter des menschlichen Lebens sind in der spätmodernen Lebenshaltung stark in den Hintergrund getreten. Damit ist es schwierig geworden, das Aufkommen von unkontrollierbarer Erkrankung zu ertragen. Krankheit kommt einem Versagen des menschlich-medizinischen Potenzials gleich. Die Bedrohung menschlichen Lebens durch das Virus CoV‑2 setzt dieser Sichtweise von der Verfügbarkeit und Sicherheit gesunden, gelingenden Lebens eine klare Grenze – und erscheint so den Menschen in zweifacher Hinsicht als Angriff und Provokation, denn Cov‑2 gefährdet die mentale wie die physische Verfasstheit. Die Vorstellung vom ungeheuerlichen und überraschend angreifenden Gegner erscheint als das passende Bild, der Aufruf zu Verteidigung und Kampf sind die metaphorische Antwort.

### Die Welt ist schuldig: Deutungen von Covid-19 als Mahnung und Strafe

Wie jegliches Erleben von Erkrankung oder Unfall Fragen nach der Ursache provoziert (Warum kam es dazu? Warum ich? Warum nicht jemand anderes? Warum jetzt?), entfacht auch Covid-19 eine Suche nach Gründen. In der Berichterstattung hierüber ist angesichts der Pandemiesituation die Welt als Ganze im Blick. Die Ausbreitung des Virus ist der Geschwindigkeit und den Verkehrswegen der global vernetzten Welt gefolgt, so dass sie inzwischen als ganze infiziert erscheint und die Symptome von Covid-19 zeigt.^12^ Im Kontext der Ursachensuche begegnet das mythisch-religiöse Bild von Sünde und Strafe. Die rasante Verbreitung von CoV‑2 führt uns mahnend die riskante Seite der Globalisierung vor Augen: Hier wehrt sich die überstrapazierte und ins Ungleichgewicht geratene Natur gegen weitere Ausbeutung, indem sie selber zur Gefahr wird.^13^ Unter den von Menschen geschaffenen Bedingungen des globalisierten Marktes mutierte als Strafe ein ursprünglich tierisches Virus und wird deren Betreibern gefährlich. Es geht nicht um Schuldzuweisung an betroffene Einzelpersonen mit Verweis auf ihre gesundheitliche Selbstverantwortung. In der Pandemie wird die Menschheit in ihrer Kollektivität adressiert und als globale Gemeinschaft schuldig gesprochen. Zudem bringt die hohe Infektiosität des Virus und die hiermit verbundene soziale Verantwortung in der Prävention den Menschen ihre Relationalität verstärkt zu Bewusstsein. Mit der weltweiten Isolierung und wirtschaftlichen Regression sühnt sie nun metaphorisch die politisch-ökonomischen Auswüchse der kapitalistischen Leistungsgesellschaften mit ihrer Gier und Notwendigkeit zu beständigem Wachstum und Markterschließung: „Coronavirus: Die Seuche als Strafe Gottes.“^14^

Wenn das Denken über Gesundheit und gelingendes Leben dominiert wird vom Glauben an deren Kontrollier‑, Plan- und Machbarkeit, dann muss eine unvorhergesehene sowie unklare und schwer steuerbare Erkrankung wie Covid-19 als willkürlich und befremdend, ja sinnwidrig erscheinen. Das provoziert die Forderung nach einer Erklärung. Hier ist der Punkt, an dem die Aufladung der Erkrankung mit Moral beginnt: Unter der Vorstellung von Krankheit als Versagen ist die Schuld bei den Menschen selbst zu suchen, d.h. angesichts der Pandemiesituation dem Kollektiv der global vernetzten Menschheit. Mit der Strafmetaphorik wird das epidemiologische Faktum, dass die Infrastruktur der globalisierten Welt die Verbreitung des Virus begünstigte, überhöht durch das Erklärungsmotiv der Mahnung gegenüber der Lebensweise der modernen Gesellschaften, die allzu expansiv und rücksichtslos über die Welt verfügen. In diesem Sinne schrieb schon Sontag: „Nichts ist strafender, als einer Krankheit eine Bedeutung zu verleihen – da diese Bedeutung unausweichlich eine moralische ist. Jegliche gewichtige Krankheit, deren Kausalität im Dunkel liegt und deren Behandlung wirkungslos ist, wird tendenziell mit Bedeutsamkeit aufgeblasen.“ (Sontag 2016: 51). „Und die Krankheit wird (solchermaßen mit Bedeutung aufgeladen) auf die Welt projiziert [...]. In der Vergangenheit wurden solche bombastischen Phantasien regelmäßig an epidemische Krankheiten geknüpft, an Krankheiten, die ein kollektives Unglück waren.“ (Sontag 2016: 52) Das kollektive Unglück der Pandemie mit Covid-19 reaktiviert diese historisch bewährten Deutungsmuster. Neben diesem historischen Hintergrund bietet die beschriebene Einstellung von Verfügbarkeit und Kontrollierbarkeit von Gesundheit und Erkrankung ebenfalls eine Erklärung für diese Verknüpfung: die hohe Gewichtung der Selbstverantwortlichkeit führt im Umkehrschluss im Krankheitsfall zur Schuldzuweisung an die Betroffenen.

### Die Welt in der Krise: Covid-19 als Krise und Tor in eine geläuterte Weltwirklichkeit

Die Zeit der Pandemie, d.h. die Zeit bis zur Überwindung des Gegners und dem Verbüßen der Strafe, wird aufgrund ihres Ausmaßes als tiefgreifender Einschnitt wahrgenommen, die die Lebenswirklichkeit der Menschen nachhaltig verändern wird. In dieser globalen Zäsur wird CoV‑2 auf seinen Sinngehalt hinterfragt: Analog zu der Erfahrung, dass Menschen nach einer Krise durch schwere Erkrankung, Krieg oder andere Katastrophen ihr Leben verändert wahrnehmen und neu ausrichten müssen, wird der globalen Pandemie ebensolche krisenhafte Wirkung zugeschrieben. In diesem Kontext erscheint das Virus als der große Veränderer, Entschleuniger und Relativierer des bisherigen Lebens: Konstanten, die das Lebensgefühl der Gesellschaftssysteme in den Industriestaaten bis dato bestimmt haben, wie die Selbstverständlichkeit menschlicher Nähe, der Eindruck weitgehender gesundheitlicher Sicherheit auf der Basis fortschreitender medizinischer Möglichkeiten sowie die Routinen weltweiter Vernetzung, Geschwindigkeit und Erreichbarkeit wurden innerhalb weniger Wochen ausgesetzt und in Frage gestellt. In den Metaphern von Schuld und Strafe liegt die Logik von Reue und Läuterung. Dementsprechend werden Hoffnung und Appell an die Menschheit kommuniziert, diese als Chance zu nutzen und gebessert aus ihr hervorzugehen: „Der Aufbruch. Jetzt oder nie: Der Corona-Schock birgt die Chance auf eine bessere Welt.“^15^ Wenn die Menschen durch die dramatischen Auswirkungen der weltweiten Seuche die Fragilität und Angreifbarkeit der menschlichen Gesundheit, Ökonomie und Gesellschaftsordnungen (wieder) erkennen, sie akzeptieren, und konstruktiv in einen geläuterten Lebenswandel integrieren, hat sich der Sinn von CoV‑2 erfüllt, und die Welt wird gesunden. Covid-19 wird metaphorisch zum Anstoß zur Läuterung und zur Tür in eine bessere Welt.^16^

Krankheit, insbesondere schwerwiegende, birgt per se mythisch-transzendente Dimensionen, da sie die Bedingtheit und Vulnerabilität menschlichen Lebens vor Augen führt und uns mit Fragen nach Sinn und Zweck des eigenen Daseins konfrontiert. Metaphern helfen mit ihren inhärenten Deutungsmustern, Krankheiten einen Sinn zuzuschreiben und sind insofern Ausdruck von Kontingenzbewältigung: eine Erkrankung, die sich als lebensgeschichtlich sinnvoll erzählen lässt, lässt sich besser ertragen und in das menschliche Grundanliegen nach einem gelingenden (weil sinnvollen) Leben integrieren (Schneider-Flume 2002: 88). Konsequent zur Schuld- und Strafmetaphorik wird im Falle von Covid-19 die Läuterung von der globalisierten Lebensweise als Zweck der Pandemie gesehen. Hiermit rückt die Frage nach den richtigen Voraussetzungen und Bedingungen für ein gutes, systemisch verträgliches Leben in den Blick. Zum Zeitpunkt von Globalisierungs- und Klimadebatte wird Covid-19 für die Kritik an dem menschlichen Selbstverständnis und Weltverhältnis in Anspruch genommen, das der Globalisierung zugrunde liegt: eine beständige Ausweitung von Wissen, Vernetzung und technisch-ökonomischer Beanspruchung der Welt und ihrer Bewohner, die z.B. von Hartmut Rosa als Prozess der „Verfügbarmachung der Welt“ beschrieben wird.^17^ Die beschriebene Einstellung gegenüber Gesundheit als weitgehend kontrollier- und herstellbares Produkt lässt sich als ein Aspekt dieses Selbstverständnisses einordnen. In dieser Dynamik und ihrer bereits vorhandenen Kritik bietet es sich an, die unvorhergesehene grenzüberschreitende und schwer kontrollierbare Erkrankung Covid-19 metaphorisch als Vehikel zu nutzen, mittels dessen der Menschheit nicht nur strafend die Risiken ihrer Lebensweise vor Augen geführt werden soll, sondern auch deren Limitationen. Das damit geschaffene Krisenbewusstsein birgt die Chance zur Umorientierung, was der Pandemie einen höheren Sinn verleiht.

## Schluss: Jenseits der Metaphern

Metaphern organisieren bewusstes wie unbewusstes Erleben und bringen zugrundeliegende Einstellungen zum Ausdruck. Sie „eröffnen bestimmte Perspektiven, sie geben etwas zu sehen als etwas, sie rufen Affekte hervor. Sie bilden dadurch Einstellungen und leiten Handeln“ (Kurz 2009: 27). Ist diese Äußerung generell auf jegliche Kommunikation bezogen, so gilt sie auch speziell für den Gebrauch von Metaphern in der aktuellen gesellschaftlichen Verständigung über die Pandemie mit Covid-19, d.h. den hier interessierenden Umgang mit Krankheit und Gesundheit. Die Berichterstattung in den öffentlich zugänglichen Medien ist ein wesentliches Verbreitungsfeld von Information wie Interpretation zu CoV‑2 und Covid-19. Indem hier Metaphern, die in wissenschaftlicher wie gesellschaftlicher Kommunikation zu CoV‑2 und Covid-19 bereits kursieren, zitiert und transportiert werden, ggf. aber auch bestimmte Sprachbilder bewusst ausgewählt oder ggf. neu konstruiert und intentional im Sinne von Interpretationen eingesetzt werden, spiegeln und prägen sie als Konstrukte kognitiver Verstehens- und Verarbeitungsprozesse retrospektiv, situativ und antizipatorisch individuelle wie gesellschaftliche Sicht- und Verhaltensweisen im Umgang mit dem Virus und seinen Auswirkungen auf die Lebenswelt. Die Auseinandersetzung mit in den Medien verwendeten Metaphern kann dementsprechend hilfreich und sinnvoll dafür sein, nicht nur individuelle oder gesellschaftlich dominierende Denkmuster und damit verbundene Verhaltensweisen bewusst zu machen, sondern diese auch kritisch zu reflektieren sowie ggf. umzudeuten und aktiv zu verändern.

Susan Sontag zielte in ihrer Auseinandersetzung mit Krankheit als Metapher auf eine Bewusstmachung der in ihren Augen problematischen, ungesunden Auswirkungen des Metapherngebrauchs im damaligen psychosozialen Umgang mit Krankheit und Kranken ab. Ihr Essay liest sich als ein Aufruf, die etablierten Denkweisen zugunsten einer ehrlichen, gesunderen, d.h. möglichst metaphernfreien Verständigung über Krankheit aufzubrechen. Wie in Abschnitt 1.2 skizziert wurde, wohnt Metaphern im Kontext der Medizin mit ihren sowohl verständnisermöglichenden als auch -formenden Implikationen allerdings ein Potenzial inne, das in seinen Auswirkungen nicht zwingend destruktiv sein muss. Vielmehr können, je nach Konnotation und Sinngehalt, unterschiedliche, d.h. krankheitsverstärkende, aber auch gesundheitsförderliche, zukunftsöffnende Konsequenzen angestoßen werden. Damit geht für alle Verwendungen von Metaphern im Zusammenhang mit Gesundheit und Krankheit (sei es z.B. im wissenschaftlichen oder im ärztlichen Kontext, sei es in öffentlichen Medien oder politischer Rhetorik) eine hohe Verantwortung für einen hinsichtlich ihrer möglichen Auswirkungen bewussten, reflektierten Einsatz einher. Susan Sontag hat diese kritische Perspektive angestoßen und auf die Verpflichtung zur sorgfältigen Überprüfung von Krankheits-Metaphern und der ihnen zugrundeliegenden bzw. mit ihnen vermittelten Kognitionen aufmerksam gemacht. Durch die kritische Reflexion werden für Kranke wie auch ggf. für Gesunde belastende oder fragwürdige Denkmuster erkennbar, und es besteht die Chance, diese bewusst zu verändern.

Welche Einsichten ergeben sich nun abschließend im Sinne eines gesunden Umgangs mit Krankheit und Erkrankten, wenn man die Covid-19-Pandemie aus ihren Metaphern und den zugrundliegenden Kognitionen herausschält?

Die Bilder um Covid-19 beziehen sich auf die kollektive Situation der Pandemie, die moralische Aufladung gilt der Weltbevölkerung in ihrer globalisierten Lebensweise. In den Metaphern wird Empörung und Hilflosigkeit ebenso transportiert wie moralisch motivierte Erklärung und Kritik an der bisherigen Lebenshaltung. Mit dem enormen Aufwand der temporären Umorganisation der Welt nach Maßgabe der Infektionsmedizin bemühen und demonstrieren die Menschen der spätmodernen Gesellschaften einerseits konsequent ihren von Verfügbarkeitsdenken und Kontrollstreben geprägten Umgang mit Gesundheit und Krankheit in seiner Funktionalität, andererseits nehmen sie auch dessen Grenzen wahr, auf physischer wie mentaler Ebene. Das führt direkt in die kritische Auseinandersetzung mit der bisherigen Lebensweise und deren Verhältnismäßigkeit. In dieser Kollision mit dem durch die Pandemie empfundenen Limitations- und Kontingenzerleben wird die metaphorische Überhöhung in den Kategorien von Strafe, Sühne und Läuterung herangezogen. In der Reflexion dieser Deutungsmuster treten die konstitutionellen Gegebenheiten von Vulnerabilität und Relationaliät des menschlichen Lebens wieder stärker vor Augen und decken die Unverhältnismäßigkeit einer Totalisierung von Verfügbarkeitsdenken und übermäßigen Ansprüchen an Sicherheit und Planbarkeit von menschlichem Leben und Gesundheit auf.

Covid-19 jenseits der Metaphern ist aus medizinischer Sicht beschreibbar als rein biologisch-physisches Phänomen einer viral bedingten, hoch ansteckenden Infektionskrankheit, für die bisher weder Pathomechanismen noch Suszeptibilitäten exakt geklärt, noch spezifische Präventiv- oder Kurativtherapeutika gefunden sind. Die kritische Reflexion und Herausschälung des Pandemiegeschehens aus ihrer Metaphorik bringt zwei Einsichten wieder verstärkt in das Bewusstsein und damit Anleitung für einen gesunderen, mythenfreien Umgang mit Gesundheit und Krankheit: Krankheiten sind weder im Sinne von ,Machsal‘ als reines Produkt menschlicher Aktion, noch im Sinne von Schicksal als unabänderlich und notwendig ,von oben‘ Gegebenes und Hinzunehmendes aufzunehmen, der Mensch weder als rein passives Opfer noch exklusiv verantwortlicher aktiver Macher von Krankheit. Vielmehr geht es um die Frage der Verhältnismäßigkeit der Position.^18^ In der Individualperspektive auf jeden einzelnen Menschen ist trotz aller Fortschritte und Kontrollmöglichkeiten der modernen Hochleistungsmedizin aufgrund der universell gegebenen, individuell je unterschiedlich ausgeprägten konstitutionellen Vulnerabilität keine Vollverfügbarkeit oder Garantie für Gesundheit und Leben zu erreichen. Als verhältnismäßig erscheint die Haltung einer steten Sensibilität für die Grenzen der Machbarkeit, die sich nicht als Schwäche oder Makel, sondern Realitätsnähe versteht. So können die Ursachenklärung, Abwehr und Bewältigung von Krankheit im Bereich des Machbaren konstruktiv und jenseits demomoralisierender Schuldfragen angegangen werden.

Daneben besteht in der systemischen Perspektive auf den Menschen als soziales, relationales Wesen unveräußerlich der Zusammenhang zwischen individuellen und kollektiven Bedingungen für gesundes Leben. Die Pandemie führt die weltweite, gemeinschaftliche Vernetzung von Gesundheitsverantwortung vor Augen. „Dass die Gesundheit jeder einzelnen Nation von der aller anderen abhängt, ist kein frommer, leerer Wunsch, sondern ein epidemiologisches Faktum.“ (Lederberg 1958, zitiert bei Wagner 2010) Die Entstehung und Verbreitung von Erkrankungen hängen unmittelbar zusammen mit der Gestaltung des menschlichen Lebensraumes, was Einflussnahme ermöglicht und gemeinschaftliche Verantwortung bedeutet.

So kann die kritische Auseinandersetzung mit der Metaphorik um Covid-19 zu einer Haltung gegenüber Krankheit und Gesundheit führen, in der die genannten konstitutionellen Gegebenheiten menschlichen Lebens wieder mehr Berücksichtigung finden: grenzsensible Wahrnehmung von Krankheit als ein sozial verantworteter Spielraum zwischen Aktivität im Bereich des Möglichen sowie Akzeptanz im Bereich des vorgegeben Notwendigen.

## Footnotes

1 *Illness as Metaphor*, in diesem Beitrag verwendet in der Übersetzung von K. Kersten und C. Neubaur: Sontag (2016). Die Verweise im Folgenden beziehen sich auf diese, also die 4. Auflage.

2 Hierbei wird ein stichprobenartiges Vorgehen zugrunde gelegt, in dem eine Auswahl online abrufbarer Medien auf wiederholt und prominent auftauchende Metaphern im Zusammenhang mit Covid-19 untersucht wurde. Es wird somit weder von Repräsentativität noch Vollständigkeit der verwendeten Medien und der im Folgenden vorgestellten Metaphern ausgegangen. Cipolletta und Ortu (2020) präsentieren in ihrer Untersuchung psychologischer Konstrukte im Umgang mit der Pandemie hinsichtlich der in der Erfahrung und Kommunikation von Covid-19 begegnenden Metaphern ein vergleichbares Vorgehen und Ergebnis.

3 Vgl. z. B. folgende Zitate in den Medien: „Trump über das Coronavirus: ‚Der schlimmste Angriff, den wir jemals hatten ‘ “. (AFP in faz-net am 06.05.^2020^); „Fünf vor acht. China und das Coronavirus. Schleichender Angriff.“ (Klingst in ZEIT ONLINE am 11.05.^2020^); Corona Forschung aktuell. Angriff auf Herz und Hirn: Wie das Corona-Virus im Körper wütet (Zenker in eSüdwestpresse am 27.07.^2020^). Vgl. darüber hinaus Marron et al. (2020).

4 Vgl. z. B. folgende Zitate in den Medien: Corona-Krise in Russland: „Es ist wie in einem Krieg“ (C. Nagel in tagesschau.de am 27.05.^2020^); „Russischer Impfstoff gegen Virus: Der kalte Corona-Krieg“ (taz.de am 13.08.^2020^); „Die ganze Welt im Krieg gegen das Coronavirus“ (Kufner in *Die Presse* am 15.04.^2020^); „Ärzte führen ‚Krieg gegen Coronavirus und Dummheit‘“ (n-tv.de am 12.07.^2020^).

5 Vgl. z. B. folgende Zitate in den Medien: „Kampf gegen Coronavirus. Wie Bundeswehr und Kirchen helfen wollen“ (tagesschau.de am 15.30.^2020^); „China zeigt, dass die Maßnahmen im Kampf gegen COVID-19 auf die örtlichen Umstände zugeschnitten werden müssen“ (Interview mit Gauden Galea, Repräsentant der WHO in China, am 03.04.^2020^); „Kampf gegen Corona: Größtes Hilfspaket in der Geschichte Deutschlands“ (bundesfinanzministerium.de am 22.05.^2020^).

6 In welcher Weise aus psychoanalytischer Sicht die Metapher vom Eindringling Abwehrmechanismen auslöst, macht z.B. Heimerl (2020) deutlich.

7 bild.de am 28.09.^2020^.

8 Vgl. z. B. folgende Zitate in den Medien: „Der Kampf hat begonnen. Wie gut Deutschlands Kliniken für den Corona-Ansturm gerüstet sind“ (SPIEGEL 2020b); „Corona besiegen wir nur weltweit – oder gar nicht“ (Müller in welt.de am 07.04.^2020^); „Corona-Forschung in Hamburg und Lübeck: ‚Forschung ist der Schlüssel im Kampf gegen das Coronavirus‘“ (bmbf.de am 14.07.^2020^).

999 Der Beitrag wurde vor Zulassung eines Impfstoffes erstellt und beschreibt daher eine andere Situation als die zum Zeitpunkt der Publikation gegebene.

9 Vgl. z.B. folgende Zitate in den Medien: „Das Virus ist unser Gegner.“ (Ackermann in psychologie-heute.de am 06.04.^2020^); „Kampf gegen einen unsichtbaren Gegner“ (von Altenbockum et al. in faz-net am 26.02.^2020^); „Viren, die unbekannten Feinde“ (Dilcher in stimme.de am 03.04.^2020^).

10 Daneben bietet die Kampfesmetaphorik gesundheitspolitisch die Möglichkeit, Helden zu schaffen, die sich in der zu schlagenden Schlacht als besonders tragisch oder erfolgreich qualifizieren. Insofern ist diese Metapher zusätzlich Ausdruck von sowohl der empfundenen Hilflosigkeit – vgl. z.B. Medienzitate wie „Coronavirus-Held ist nun selber tot - er ließ nicht zu, dass alles vertuscht wird“ (merkur.de 2020) oder „Li Wenliang - Ein tragischer Held“ (Sieren in dw.com am 01.03.^2020^) oder „Retter in der Coronakrise: Stille Helden“ (Weiss und Bergmann in faz-net vom 11.05.^2020^) – als auch der Hoffnung auf Rettung, z.B. in Gestalt von Mediziner*innen oder Politiker*innen, die von dieser Rolle und Pose zum Handeln angetrieben werden. Vgl. z.B. Medienzitate wie „Im Kampf gegen Corona will die Deutsche Post zum Impfstoff-Helden werden“ (Nicolai in welt.de am 05.08.^2020^), oder: „Kampf gegen Corona: Dieser Mann kann zum Helden werden“ (Harms in t‑online vom 07.04.^2020^).

11 Entgegen dem weit verbreiteten Wunsch nach einem Sterben im vertrauten Umfeld zu Hause sterben gemäß den Erhebungen des Statistischen Bundesamtes von 2011 rund 75 % der deutschen Bevölkerung in medizinisch geprägten Einrichtungen wie Kliniken, Senioren- und Pflegeheimen, Hospizen. Krankenhäuser sind mit über 50 % die häufigsten Sterbeorte, zu Hause versterben 23 %. Vgl. Borasio 2013 und Dasch et al. 2015. Zu einzelnen Aspekten und Bedingungen für ein „Gutes Sterben“ vgl. Hutter et al. 2020.

12 Vgl. z.B. Medienzitate wie: „Die Welt ist krank – ein Überblick in Grafiken“ (zdf. de am 07.04.^2020^).

13 Vgl. z.B. Medienzitat wie „Made in China. Corona-Virus. Wenn die Globalisierung zur tödlichen Gefahr wird“ (SPIEGEL 2020c).

14 Seeßlen in ZEIT ONLINE am 03.03.^2020^. Vgl. z.B. weitere Medienzitate wie: „Sind Unglücke wie Corona eine Strafe Gottes?“ (Steller in efo-magazin.de am 19.08.^2020^); „Ist die Corona-Epidemie die Strafe Gottes?“ (Kajan in die-tagespost.de am 27.03.^2020^). „Corona als Strafe Gottes“ (Gensing 2020).

15 SPIEGEL 2020a. Vgl. z.B. weitere Medienzitate wie: „Wie Corona unsere Welt verändern wird – das erwartet der Seuchen-Experte“ (Hoß in stern.de am 02.05.^2020^); „Corona-Krise. Die Lichtblicke danach“ (stern.de am 07.04.^2020^).

16 Hierzu passend veröffentlichte das kalifornische Unternehmen für Künstliche Intelligenz, machineVantage, „gateway“ als die mittels Künstlicher Intelligenz identifizierte Metapher, die geeignet sei, in der Öffentlichkeit Covid-19 bedingte kritische Informationen und Veränderungen effizient zu kommunizieren (PR Newsire 2020).

17 In diesem Prozess ist die Welt primär ein Objekt, das in allen Dimensionen „gewusst, beherrscht, erobert, nutzbar gemacht werden“ muss (Rosa 2020: 12). Rosa untergliedert den Prozess der Verfügbarmachung der Welt in vier Aspekte: erkennen (sichtbar machen, was da ist), erreichen (zugänglich machen), beherrschen (unter Kontrolle bringen) und nutzbarmachen (zum Instrument unserer Zwecke machen). In dieser Perspektive erscheint den Menschen alles als Aufforderung zur Verfügbarmachung, d.h. letztlich als ein Widerstand, der zu bezwingen ist, d.h. die Welt als „Aggressionspunkt“ (Rosa 2020: 10). Aggression provoziert Gegenwehr: Nun wehrt sich die Welt.

18 „Die extreme Position einer uneingeschränkten Kalkulierbarkeit unserer effizienten Gesundheitsvorsorge ist ebenso unsinnig wie die gegenteilige Annahme einer Unverfügbarkeit unseres Gesundheits- oder Krankheitszustandes, der als gegeben hinzunehmen ist und nur minimal willentlich reguliert werden kann.“ (Lesch 2014: 191). Zu Hintergrund und Funktion der Auffassungen von Schicksal und Machsal im Zusammenhang mit Krankheit vgl. Maio 2014.
